# Practices for collecting, analyzing and disseminating data on health and its social determinants among Black populations in Quebec: a scoping review

**DOI:** 10.24095/hpcdp.45.4.03

**Published:** 2025-04

**Authors:** Nina Mombo, Kim Ngan Le Nguyen

**Affiliations:** 1 Eval-Expert Sant, Laval, Quebec, Canada; 2 Independent researcher, Montral, Quebec, Canada

**Keywords:** racialized populations, ethnoracial data, health of Black populations, collection methods

## Abstract

**Introduction:**

The COVID-19 pandemic highlighted the deficiencies in healthcare systems both within and outside of Canada, affecting racialized populations, particularly Black communities, who face an increased risk of infection and mortality from the disease. Although Black populations in Quebec make up more than 25% of the Black population in Canada, detailed data on the impact of COVID-19 on these communities are only available at the national level. This scoping review documents the methods and issues related to the collection, analysis and dissemination of data on the health of Black populations in Quebec, and its social determinants.

**Methods:**

We conducted a review of studies published in English and French from January2010 to June2024 by consulting six databases. This review exclusively comprised studies involving data collection from racialized populations, including Black populations in Quebec, and excluded Canada-wide studies involving only a subsample of Black populations in Quebec. The main keywords used were: “data on race”, “ethnic data collection”, “race data collection”, “culturally appropriate”, “health”, “survey”, “questionnaire”, “racial groups”, “racialized groups”, “Black and minority ethnic people”, “people of colour”, “migrants”, “Quebec”, “collecte de donnes”, “minorit”, “noir” and “ethnicit”.

**Results:**

We selected 43studies covering four sectors: health, social services, education and employment. We identified the main issues, methods and strategies used to recruit members of Black communities and to collect and analyze data according to ethnoracial categories while minimizing bias to better understand the sociocultural and socioeconomic context of the target populations.

**Conclusion:**

Our review highlights the importance of collecting data on racialized groups, particularly Black communities in Quebec, to support public policies aimed at promoting health equity.

**Highlights**

- Although Black populations in Quebec make up more than 25% of the Black population in Canada, detailed health data for these populations in Quebec are lacking.
- We analyzed 43 studies published since 2010 that focussed on research conducted among Black populations in Quebec.
- We identified key issues, methods and strategies used to recruit members of Black communities in Quebec, and to collect and analyze data according to ethnoracial categories, while limiting bias to better understand the realities of these populations.
- The results of this scoping review could support public policies focussed on these populations to promote health equity.

## Introduction

Collecting ethnoracial data on health and its social determinants raises complex ethical issues.^1^ The concept of “race” has no basis in biology (which is why we have chosen to set it in quotation marks throughout this paper), but has long been used as an excuse for discrimination. This fact in turn has led to hesitation in or misperceptions about collecting ethnoracial data in Canada, particularly in Quebec.^2^-^4^ However, these data are crucial for measuring health inequalities and establishing informed public health policies. 

For example, Statistics Canada data show that Black Canadians had the highest number of deaths due to COVID-19.^5^ In Quebec, the COVID-19 mortality rate was three and a half times higher in neighbourhoods with at least 25% racialized populations.^5^ Although the Black population in Quebec makes up more than 25% of the Black population in Canada, accurate data on the impact of COVID-19 on Black communities at the provincial level are lacking, and available only at the national level.^5^,^6^ A report by the Direction rgionale de sant publique deMontral (the public health authority in Montral) provides information on the COVID-19 situation in Montral, but lacks data analysis by ethnic origin, despite the concentration of cases in the region, which has the largest Black population in the province of Quebec.^7^ In contrast, Quebec researchers emphasize the importance of collecting health data based on ethnoracial background.^8^

Compared with the United States and the United Kingdom, Canada has limited experience with the systematic collection of ethnoracial health data within provincial health systems.^9^ However, early initiatives have been rolled out in Eastern Canada, Ontario^10^ and Nova Scotia,^11^ though their implementation remains difficult and raises significant issues.^2^,^3^

Despite existing efforts, there is no consensus in Canada or the United States on the standards for collecting ethnoracial and sociodemographic data.^12^,^13^ How to define the identity of racialized populations, particularly Black populations, is still under debate.^14^-^17^ Additionally, the most relevant social determinants of health for understanding health equity issues of Black populations in Canada, and particularly in Quebec, are not clearly defined, nor is the method for obtaining these data.^18^,^19^

Throughout the COVID-19 pandemic, the Institut national de sant publique du Qubec (INSPQ) and the regional public health authorities (particularly in Montral) collected data by age, gender and territory or borough of residence.^7^,^20^ These data indirectly indicate a higher number of COVID-19 cases in areas of Montral with a greater proportion of visible minorities.^7^,^21^ However, Quebec still lacks systematically collected health data based on ethnoracial background. The purpose of this article is to document the methods and issues involved in collecting, analyzing and disseminating data on the health of Black populations in Quebec, and its social determinants.

We conducted a scoping review to identify studies dealing exclusively with Black populations or racialized groups, including Black populations in Quebec, to analyze the methods and issues faced by researchers. 

## Methods

Scoping reviews “aim to map rapidly the key concepts underpinning a research area and the main sources and types of evidence available, and can be undertaken as stand-alone projects in their own right, especially where an area is complex or has not been reviewed comprehensively before.”^22^^,p.194,^^23^ This review was exploratory and non-exhaustive, providing an overview of the extent of research in the field of health and its social determinants as they pertain to Black populations in Quebec.^24^ We applied the PRISMA-ScR approach (Preferred Reporting Items for Systematic Reviews and Meta-Analyses Extension for Scoping Reviews) used by Tricco et al.^25^

***Review of studies***

The review of studies was carried out in two stages: an initial review in September 2022 and an update in June2024. We conducted an initial literature search on studies published in English and French between January2010 and September2022 by consulting the PubMed, Embase, Social Care Online, rudit, CAIRN and CINAHL databases. The main keywords used were: “data on race”, “ethnic data collection”, “race data collection”, “culturally appropriate”, “health”, “survey”, “questionnaire”, “racial groups”, “racialized groups”, “Black and minority ethnic people” (which includes “African Caribbean people” and “African people”), “people of colour”, “migrants”, “Quebec”, “*collecte de donnes*”, “*minorit*”, “*noir*”, and “*ethnicit*” (Table1). The update was carried out on 3June2024 on Google Scholar using the keywords “race-based data collection,” “Black” and “Quebec”. We consulted the first 20pages of results. Despite the subjectiveness inherent in this search engine, past scoping reviews and systematic reviews have used it and justified its use.^26^-^28^

**Table 1:** Research strategy (partial list)

Database	Search terms
**PubMed**	(health[Title/Abstract] OR healthcare[Title/Abstract] OR hospital*[Title/Abstract]) AND ("Ethnicity"[Majr] OR "Racial Groups"[Majr:NoExp] "Black*" OR "African*" OR "Caribbean*" OR "Afro*" OR "Person of colo?r*" OR "People of colo?r*" OR "colo?red*" OR "African*"dark-skin*” OR "ethnic minorit*") AND ("Data Collection/methods"[Majr:NoExp] OR "Data Collection/organization and administration"[Majr:NoExp] OR "Data Collection/standards"[Majr:NoExp] OR "Data Collection/statistics and numerical data"[Majr:NoExp] OR "Focus Groups"[Majr] OR "Records/methods"[Majr] OR "Records/organization and administration"[Majr] OR "Records/statistics and numerical data"[Majr] OR "Records/supply and distribution"[Majr] OR "Surveys and Questionnaires/methods"[Majr:NoExp] OR "Surveys and Questionnaires/organization and administration"[Majr:NoExp] OR "Surveys and Questionnaires/standards"[Majr:NoExp] OR "Surveys and Questionnaires/statistics and numerical data"[Majr:NoExp] OR "culturally tailored"[Majr] OR "cultural competent"[Majr] OR "culturally targeting"[Majr] OR "culturally appropriate"[Majr]) Filters: from 2010–2022 (("ethnicity data"[Title/Abstract] OR "race data"[Title/Abstract] OR "data on race"[Title/Abstract] OR "ethnic data"[Title/Abstract]) AND (collect[Title/Abstract] OR collection[Title/Abstract] OR collecting[Title/Abstract] OR gather*[Title/Abstract] OR monitor*[Title/Abstract] OR questionnaire*[Title/Abstract] OR survey*[Title/Abstract]) AND (health[Title/Abstract] OR healthcare[Title/Abstract] OR hospital*[Title/Abstract]) AND (Quebec)[Title/Abstract] OR (Qubec)[Title/Abstract])) Filters: from 2010–2022
**CAIRN**	"collecte de donnes" W/5 ethnique; limite=10 ans; "ethnic data" W/5 monitor*; limite=10 ans "collectes de donnes" W/5 ethnique; limite=10 ans; "ethnic data" W/5 survey*; limite =10 ans "collecte de donnes" W/5 race; limite=10 ans; "ethnic data" W/5 questionnaire*; limite=10 ans "collectes de donnes" W/5 race; limite=10 ans; "ethnicity data" W/5 collect*; limite=10 ans "collecte de donnes" W/5 minorit*; limite=10 ans; "ethnicity data" W/5 gather*; limite=10 ans "collecte de donnes" W/5 ethnicit; limite=10 ans; "ethnicity data" W/5 monitor*; limite=10 ans "collectes de donnes" W/5 ethnicit; limite=10 ans; "ethnicity data" W/5 captur*; limite=10 ans "collecte de donnes" W/5 noir*; limite=10 ans; "ethnicity data" W/5 captur*; limite=10 ans "collectes de donnes" W/5 noir*; limite=10 ans; "ethnicity data" W/5 survey*; limite=10 ans "collecte de donnes" W/5 haitien*; limite=10 ans; "ethnicity data" W/5 questionnaire*; limite=10 ans questionnaire* W/5 ethnique; limite=10 ans; "race data" W/5 collect*; limite=10 ans questionnaire* W/5 race; limite=10 ans; "race data" W/5 gather*; limite=10 ans questionnaire* W/5 minorite; limite=10 ans; "race data" W/5 survey*; limite=10 ans questionnaire* W/5 ethnicit; limite=10 ans; "race data" W/5 questionnaire*; limite=10 ans sondage* W/5 ethnique; limite=10 ans; "data on race" W/5 collect*; limite=10 ans sondage* W/5 race; limite=10 ans; "data on race" W/5 gather*; limite=10 ans sondage* W/5 minorite; limite=10 ans; "data on race" W/5 captur*; limite=10 ans sondage* W/5 ethnicite; limite=10 ans; "data on race" W/5 monitor*; limite=10 ans "ethnic data" W/5 collect; limite=10 ans; ethnic W/5 data W/5 monitor*; limite-10 ans "ethnic data" W/5 gather*; limite=10 ans; ethnicity W/5 data W/5 collec; limite-10 ans "ethnic data" W/5 captur*; limite=10 ans; race W/5 data W/5 collect*; limite-10 ans race W/5 data W/5 captur*; limite-10 ans race W/5 data W/5 survey*; limite-10 ans

***Selection of studies***

The two authors (NM and KNLN) independently selected the studies using the Population, Concept and Context (PCC) method^29^ based on the following criteria:

- Population: people from Black (skin colour) communities of all ages living in Quebec, regardless of whether they are Anglophone, Francophone or allophone;
- Concept: primary studies focussing exclusively on Black populations in Quebec, or on other racialized groups in Quebec including Black populations in Quebec, in the field of health and its social determinants (employment, education and access to social services); and
- Context: studies conducted between 2010 and 2024 in various settings in Quebec (schools, nonprofit organizations, hospitals, etc.) and published in English or French.

The exclusion criteria were as follows:

- studies involving Black populations in various Canadian provinces in which Black populations in Quebec formed only a subsample;
- studies including “race” or ethnicity as variables that did not involve analysis or reporting of data according to these variables;
- qualitative studies with fewer than five Black participants;
- studies dealing with Black populations in relation to feminism, racial profiling, the criminal justice system or the sociology of racism;
- studies that focussed specifically on the study of sexual diversity in Black populations; and
- reviews, opinion pieces, editorials and organization reports (grey literature).

Once the selection of studies had been completed, the two authors (NM and KNLN) met to review the list based on the previously established inclusion and exclusion criteria. Any discrepancies in the selection were resolved by consensus between the two authors. Additionally, the bibliography of the selected studies was reviewed by both authors to improve the literature search.

***Data extraction***

Two data extraction tables were developed and used. The following information was recorded for quantitative studies: first author, year, title, location, population, period (or study duration), purpose of study, data collection methods and study design, recruitment methods, variables collected, primary and secondary endpoints, types of analysis, main results, key findings and sources of funding (Table2). The following information was added for qualitative and mixed studies: theoretical framework applied to data analysis, main topics addressed in the interviews and consent process. The data extracted from the studies have been verified by the two authors (NM and KNLN). Given the exploratory nature of this scoping review, the quality of the selected studies was not assessed.^22^

**Table 2:** Data extraction table

Authors (year)	Purpose of the study	Population and period	Method and funding	Main results	Findings/implications
Adeponle et al. (2012)^70^	Assess the impact of using the DSM‑IV‑TR cultural formulation to diagnose psychotic disorders among patients of ethnic minority^a^ and immigrant backgrounds	323 patients from the cultural consultation service, Montral Jewish General Hospital 1999–2009	Quantitative Funding: NR	A total of 34/70 cases with a referral diagnosis of a psychotic disorder were rediagnosed as nonpsychotic, while 12/253 cases identified as nonpsychotic received a new diagnosis of a psychotic disorder. Receiving a new diagnosis of a psychotic disorder was significantly associated with being a recent immigrant (OR = 6.05), being non-Black (OR = 3.72) and having been referred by professionals other than physicians (OR = 3.23).	While the results highlight the clinical utility of the cultural formulation to improve diagnostic accuracy, the specific aspects of the cultural consultation that contribute to this outcome remain unknown.
Arcand et al. (2016)^68^	Explore the academic perseverance of permanent resident students in Montral	426 university students in Montral 2009–2010	Mixed Funding: FRQ–Socit et culture	Among the respondents, 40% found it difficult to establish interactions among ethnic groups. Students had difficulty establishing ties with the majority group. Most focus group participants felt that their isolation was mainly due to their difficulties in French. Some were thinking of moving to another province to find a job that matched their training.	Given the apparent inequalities in terms of intergroup relations, returning to university might be detrimental to their stated goal of rapid and successful integration into the host society, particularly the labour market. Returning to university in a migratory context might have a demoralizing effect, since the experience foreshadows the systemic barriers that these students may encounter in their efforts to enter the labour market.
Auger et al. (2012)^30^	Measure perinatal health outcomes among Haitian women and assess modifying factors in terms of severity	2 124 520 births in Quebec 1982–2006	Quantitative, retrospective Funding: NR	Compared with Canadian‑born mothers, the risk for Haitian‑born mothers was 4 times higher for extreme preterm birth, 2 times higher for very preterm birth and 25% higher for moderate preterm birth.	Haitians in Quebec may be a particularly vulnerable group, as they are exposed to poor health outcomes. Additional efforts are needed to assess the health status of the Haitian community and of other minority communities to determine whether other health disparities exist.
Beauregard (2020)^64^	Analyze ethnic relations between Quebeckers in majority and minority situations in terms of access to employment	1569 resums, 523 job offers in Qubec City January to July 2018	Quantitative Funding: NR	While female gender decreases discrimination, the analysis reveals an ethnic‑gender hierarchy and significant variations among minorities. Racialized women are invited to interviews more often than their male peers.	While the ratio for the Latin American female candidate suggests a lack of discrimination against her, the indicators observed for the Arab and Black male candidates suggest that the latter experience more unequal treatment in Qubec City than in Montral.
Boatswain-Kyte et al. (2020)^58^	Examine racial inequalities related to services from the DPJ in Quebec	15 875 Anglophone children aged under 15 y and 4382 children aged under 17 y reported to the DPJ 2002–2011	Quantitative, retrospective, longitudinal Funding: SSHRC, FRQ—Socit et culture	Black children’s records were reviewed, corroborated, and brought to court 5 times more often than the records of White children. Black children were also 5 times more likely than White children to be placed in foster care. The protection reports of children from other visible minorities^a^ were screened in twice as often as those of White children. The inequality rate has gradually decreased over 10 years for other visible minorities^a^ but has continued to rise for Black children.	By partnering with communities, child protection services can help support the infrastructure needed to build capacity, improve service coordination and strengthen the community resilience needed to improve outcomes for Black children.
Boatswain-Kyte et al. (2022)^59^	Examine the results of reunifications of Black children following placement in out-of-home care	1395 children received services from Quebec’s DPJ 2002–2011	Quantitative, retrospective, longitudinal Funding: SSHRC, FRQ—Socit et culture	Black children spend longer periods of time in out-of-home placement and are less likely to experience family reunification compared with other children. Poorer reunification outcomes for Black children are associated with placement instability, the age of the child and reasons for child welfare involvement.	Racial disparities in reunification vary depending on a combination of factors that are unique to the child, their family and the DPJ, as well as the family’s external factors. When younger children are reported, being Black significantly reduces the likelihood of reunification. Thus, being Black only leads to inequalities in the presence of other factors.
Boudarbat et al. (2010)^65^	Establish the professional profile of immigrants to Quebec	1875 economic immigrants admitted to Quebec 1997–2000	Quantitative Funding: NR	There is a strong correlation between country of origin and the extent to which employment matches expectations for both men and women. Men from Eastern Europe, Africa (including the Maghreb region) and Western Asia are the least likely to be satisfied with their jobs. Among African women, there is a very strong tendency to occupy positions that fall short of their expectations.	The public policy implications of these results relate to the following: (1) the issue of resources dedicated to recruiting and providing information to immigrants in their country of origin; (2) the efforts that the host community must make to help them enter the job market; (3) the need to adjust the selection grid to increase the chances of success of newcomers.
Brousseau et al. (2021)^69^	Explore factors associated with SARS‑CoV-2 seroprevalence in healthcare workers during the first wave of the pandemic	2056 healthcare workers from 10 Quebec hospitals July to September 2020	Quantitative, cross‑sectional, prospective Funding: MSSS	Of the 2056 healthcare workers, 11.7% were seropositive for SARS-CoV-2. The incidence of seropositivity was significantly higher among Black individuals and Latin Americans than White individuals, with an increased risk of 41%. The most exposed workers (support staff) had an increased risk of at least 30%.	The healthcare workers who were the most directly and most frequently in contact with patients were the most affected by COVID-19. Being Black or Latin American was associated with seropositivity. The high risk of SARS‑CoV-2 infection among healthcare workers requires making vaccination among this category of workers a priority.
Carazo et al. (2022)^54^	Measure the prevalence of psychological distress among Quebec healthcare workers, whether or not they were infected with SARS‑CoV-2	4068 patients + 4152 controls 2020–2021	Quantitative Funding: MSSS	The prevalence of high work-related psychological distress was 42%. It was associated with risk factors such as work-life balance, value conflicts and high psychological demands but not associated with SARS-CoV-2 infection. COVID patients were more often men, older, identified as Black, and worked more often as patient healthcare assistants and in long-term care facilities.	Primary prevention measures targeting psychosocial risk factors are needed to reduce mental health risks for healthcare workers.
Collins et al. (2018)^39^	Study the postsecondary pathways of Montral youth of Haitian origin	11 Quebec students of Haitian origin Period: NR	Qualitative Funding: NR	The students’ pathways were marked by various financial, institutional and social barriers. They had negative experiences with guidance counsellors during the transition to postsecondary education level. Perceived racism and discrimination emerged as themes in the young people’s discourses.	The study provides complex insights on some aspects that may be hidden in studies that traditionally group Haitians into broader categories, such as “Caribbean,” “Black” or “immigrant.”
Dagher et al. (2024)^66^	Describe the experience of access to care and access to interpreters during admission to four Montral hospitals	1104 hospitalized patients who tested positive for SARS‑CoV‑2 March to June 2020	Quantitative Funding: Gilead, the Jewish General Hospital Foundation	There were 36% of immigrants with a language barrier who did not have access to an interpreter during hospitalization. Prior to admission, 14/41 of allophone immigrants had difficulty accessing COVID-19 information in their first language. Among non‑White allophone immigrants, 9/27 had difficulty accessing COVID-19 services.	A large proportion of patients had difficulty accessing COVID-19 information and services, which may have increased exposure to SARS‑CoV‑2 and hospitalizations. After hospitalization, a large proportion of them did not have access to interpreters. Providing information and care in the first language of these communities is important for promoting health equity.
Darwish et al. (2022)^56^	Describe the characteristics of healthcare workers admitted to hospital with COVID-19 and associated risk factors for ICU admission and death	150 healthcare workers admitted to four Montral hospitals between 1 March and 30 June, 2020	Quantitative, retrospective Funding: MSSS	Migrants made up 68% of hospitalized workers, with sociodemographic characteristics that were similar to those of Canadian-born workers. Immigrants were more likely to be personal support workers than their Canadian‑born colleagues and more likely to be Black. Over 1/3 of workers had not received COVID-19-specific infection control training and over 50% did not always have access to personal protective equipment.	The results of this study are similar to those of a survey conducted by the INSPQ between May 2020 and May 2021, in which personal support workers and foreign‑born or Black healthcare workers were 2.2 times, 1.3 times and 2.5 times more likely, respectively, to test positive for SARS-CoV-2 than other healthcare workers. These health disparities are unexplained.
Debrosse et al. (2024)^49^	Explore the links between neighbourhood experiences, the ethnic/ideal alignment of identities and well‑being in youths	179 young people who were members of racialized groups living in the east end of Montral Period: NR	Quantitative Funding: FRQ	Youths’ neighbourhoods predict the extent to which they perceive that opportunities are accessible to them. Youths who reported more opportunities for people similar to them in their neighbourhood tended to report higher alignment between their racial/ethnic and ideal future identities and higher flourishing.	The findings highlight the connection between neighbourhood factors—such as cues about whether similar people are welcomed, valued and have access to opportunities—and the identities and well‑being of Black and Indigenous youths and youths of other racialized groups.
Dufour et al. (2015)^60^	Study the relationships between neighbourhood characteristics and rates of immigrant children reported to child protective services	8263 children reported to the DPJ from 505 census areas 2009–2010	Quantitative, retrospective Funding: CIHR, Centre Jeunesse de Montral	Black children living in Montral neighbourhoods where there are low education levels, a higher rate of lone-parent families, low population density and a small number of Black children are more likely to be reported to the DPJ.	Analyzing the distribution of reporting rates without considering ethnocultural background underestimates important observable differences. This shows that services need to be adapted to the realities specific to each neighbourhood and ethnocultural group.
Fang et al. (2023)^31^	Study the relationship between discrimination and disparities in healthcare accessibility	531 Black people living in Quebec April 2021	Quantitative Funding: CIHR, SSHRC	Black Anglophone participants experienced more discrimination, had fewer healthcare providers, had less access to COVID-19 information during the pandemic and were more dissatisfied with the healthcare system than their Francophone peers.	Discrimination based on racialized identity and language is pervasive in healthcare, reinforcing greater dissatisfaction in this regard, which establishes the intersecting effects of multiple forms of discrimination as unique stressors with detrimental impacts on health.
Frounfelker et al. (2022)^50^	Study the link between social distancing due to COVID-19 and mental health	3183 people living in Quebec June 2020	Quantitative Funding: MUHC Foundation and McGill University	Five classes of individuals were identified based on the perceived aspects of social distancing related to COVID-19: “Low Impact,” “Freedom/Flexibility,” “Safety,” “Family/Home” and “Hardships.” People in the “Hardships” class (more often from racialized groups and unemployed) were more likely to report a significant impact of COVID-19 on their mental health (OR = 2.09).	The analysis identified a subgroup of individuals (the “Hardships” class) who presented a higher risk of mental health problems than the rest of the population and who could be prioritized for awareness and intervention efforts. The “Low Impact” class suggests that, for healthcare workers, normalizing an abnormal crisis can be a successful coping strategy.
Gomez Cardona (2012)^32^	Explore the testimonies of children affected by gastrointestinal disorders and those of their mothers	5 Haitian families from Montral 2008–2009	Qualitative Funding: FRQ—Sant	Six therapeutic dynamics were highlighted: (1) multiple care (illness of unknown origin); (2) nutrition and the mother’s central role (gas-related illnesses); (3) contagion and withdrawal into the family (illnesses of microbial origin); (4) social suffering; (5) medical/religious approach (prayers and maintaining ties with the community); (6) no medical consultation.	The results confirm that Haitian families’ low attendance at the gastroenterology clinic could be due to the following: (1) their ability to treat their children’s stomach aches using means other than biomedicine; and (2) the fact that stomach aches are seen as a problem that does not require a medical opinion. Mistrust of healthcare services is due to negative experiences.
Kamanzi et al. (2018)^62^	Study the postsecondary pathways of Quebec youth from immigrant backgrounds	20 387 students, including 5334 with at least one first generation immigrant parent. 1994–2004; 2002–2012	Quantitative Funding: NR	The rate of access to postsecondary education is higher among youth from East Asia (80%), the Maghreb region and the Middle East (74%), but lower among those from Latin America and the Caribbean (58%). Their access pathway is generally linear.	Despite significant differences in postsecondary education access, pathway morphology tends to be similar between immigrants when educational background in high school is accounted for: the commonality is a linear pathway to university specifically.
Kamanzi (2021)^61^	Explore the factors for academic resilience among young people of African and Caribbean origin	8415 Montral students, including 574 who were originally from the Caribbean or sub‑Saharan Africa 2003–2013	Quantitative, longitudinal, retrospective Funding: NR	Students of African and Caribbean origin attend college at a rate that is comparable to that of their peers of European-Canadian origin. Black students enrolled in college are less likely to graduate by age 22. In addition, university is less accessible to them.	Some Black Quebec students manage to overcome barriers despite the fact that they are more exposed to precarious living conditions. The author calls on public authorities to place greater emphasis on improving learning conditions and supporting success.
Kanout et al. (2014)^45^	Explore academic issues and the use of community resources by Haitian families	31 parent-student pairs 7 community workers 10 teachers, principals Period: NR	Qualitative Funding: SSHRC	The data intersects the views of students, parents and school/community stakeholders on students’ resilience factors and the specific family realities that need to be considered to better support families. Parents and workers highlighted the effects of the migration journey on parenting and the intertwined relationship between the family’s migration journey and the child’s academic plans.	A better understanding of the links between the family’s migration journey and the student’s academic plans would better support students from an immigration background and anticipate various pitfalls likely to hinder the school/family relationship and the parents’ involvement with the school. Schools would benefit from working with community groups that have expertise in supporting immigrants.
Kanout et al. (2016)^71^	Explore the academic experience of students from immigrant backgrounds	32 immigrant elementary school students 2010	Qualitative Funding: NR	The students shared their views on the variations in their identity, their relationship with learning, their academic plans and their use of spaces and resources in their neighbourhood.	An intercultural perspective at school should include the student’s social/educational experience, acknowledge the student in their identities and achievements, instill a certain heterocentric approach in the interpretation of the program, support living in harmony, manage daily school life in an inclusive way and develop a partnership with families and the community.
Kanout et al. (2020)^46^	Examine the educational persistence of permanent resident students	1077 students enrolled in six higher education institutions in Quebec Period: NR	Mixed Funding: NR	Students are worried about building a comfortable home in a new society. This comfort is tied to the financial resources available and the caregivers’ responsibility for their children. Students found it difficult to create the optimal conditions to support their family needs and balance family, studies and work.	It would be important to document the challenges relating to the transition to the workplace, social integration, balancing family/studies/work, decoding university culture and practices, and the perception of discriminatory practices.
Lafortune et al. (2020)^40^	Examine Haitian students’ relationship with their college‑level studies in Montral	34 students 11 teachers 8 other professionals 2017–2018	Qualitative Funding: NR	The college experience: [translation] “We were abandoned in the wilderness, it was a cold plunge, I hit a wall, I was fed up” (p. 23). Participants were unanimous in emphasizing the individual factors in college success (motivation, vocational choice, work habits). The student experience was affected by immigration status and minority status.	The authors reiterate the importance of shared values, such as the principle of nondiscrimination. They suggest continuing to raise awareness among stakeholders about the impact of systemic factors that contribute to the marginalization and exclusion of certain minority groups. CEGEPs share responsibility for ensuring equality of opportunity and the inclusion of all students.
Lafortune et al. (2024)^48^	Examine the practices and activities that promote French language proficiency among immigrant youth in Montral	21 elementary and high school students attending a summer educational camp 6 workers July to August 2021	Qualitative, participatory research Funding: SSHRC, UQAM	Young people and guardians reported improvements in language skills (vocabulary, oral fluency, improved reading skills, etc.). The camp gave young people with limited social networks an opportunity to expand their circle of friends.	The educational camp experience highlighted the levers for efficient community/family/school collaboration. It consolidated school/community collaboration, which allowed the partnership to be maintained for future camps and for the creation of new joint projects.
Leduc et al. (2021)^57^	Study barriers faced by Black students in entering medical school in Quebec	4283 candidates for the Doctor of Medicine program in Quebec 2019–2020	Quantitative, cross‑sectional, prospective Funding: NR	The proportion of Black students in the applicant pool for medical school in 2020 is estimated to be 4.5%. It is estimated that Black students represented 1.8% of applicants invited to admission interviews and 1.2% of admitted students in Quebec in 2019. Although no direct comparisons can be made, it seems that Black applicants are disproportionately rejected compared with non‑Black students.	Two barriers are noted: (1) a significant proportion of students from Black communities in Quebec may not consider medicine as a career and do not apply to medical school; and (2) when Black students apply to medical school, their applications seem to be disproportionately rejected before the interview compared with non-Black students. A longitudinal study is needed to monitor progress and determine the factors that contribute to this progress.
Livingstone et al. (2014)^41^	Identify actions to reduce the dropout rate among Black students	20 Black students from four Montral high schools 2009–2010	Qualitative, participatory action research Funding: NR	According to the students, their academic success was influenced by multiple and interrelated factors, including family, peers, school and neighbourhood. In their view, schools should offer more support to Black students by fostering a better school climate and introducing multicultural curricula and innovative pedagogies.	The authors recommended that participatory action research with youth be introduced in schools as a tool for enhancing school success in the long term. Young people’s insight and idealism shows that dropping out of school is not as an unsolvable issue as it might seem.
Magnan et al. (2017)^43^	Explore immigrant children’s relationship to education and their postsecondary choices	60 students from immigrant backgrounds Period: NR	Qualitative Funding: NR	Young people from the Caribbean, sub‑Saharan Africa and Latin America are more likely to follow the paths that coincide with the following approaches: [translation] “elitist/laissez-faire, laissez-faire/laissez-faire and cocoon/laissez-faire.”	Two issues deserve more attention: How can the school system’s resources be used to facilitate young people’s freedom of choice and how can they be better equipped? How can we better support families in their understanding of the school system and the job market?
Magnan et al. (2017)^42^	Understand the experience of immigrant youth in their choice of CEGEP program	60 students from immigrant backgrounds Period: NR	Qualitative Funding: NR	An analysis of testimonies from immigrant youth shows that the family plays a determining role in the choice of postsecondary programs. Compared with youth from other countries, youth from sub-Saharan Africa, the Caribbean and Latin America perceived facing more constraints (familial, economic and academic) when choosing their programs.	Youth from Asia, Eastern Europe, the Maghreb region and the Middle East are more likely to follow an academic path in line with the [translation] “elitist/supportive approach.” Youth from the Caribbean, sub-Saharan Africa and Latin America are more likely to follow a path in line with the [translation] “elitist/laissez-faire approach.” This can be explained in part by the fact that Black families are more likely to belong to low or middle social classes.
Magnan et al. (2023)^72^	Study the factors that shape the academic pathways of Black students in Quebec	12 Black students of African or Caribbean origin Period: NR	Qualitative Funding: Citizen’s forum on Black representation in health sciences	Most students first enroll in a program other than their desired one to ensure their financial security or to improve their grades for a limited-enrollment program. Medicine and pharmacy studies remain a dream for most of the participants.	Students face a seemingly unfair admissions system for highly selective programs (medicine and health sciences). These results shed light on possible changes to admissions policies for certain programs.
Mnard et al. (2020)^63^	Measure the rate of pregnancy complications in a multicultural group of women	1387 pregnant women 2013–2015	Quantitative Funding: Canadian Foundation for Dietetic Research	The risk of anemia was higher in Black women than in White women (aOR = 1.74). Black women were at higher risk of preterm birth (aOR = 1.79). Immigrant women had an increased risk of anemia compared to Canadian-born women (aOR = 1.85).	Nutritional interventions need to be targeted toward prevention of adverse pregnancy outcomes, prioritization of higher-risk groups and adaptation of the community organization’s program to a multiethnic, low-income population.
Miconi et al. (2021)^51^	Investigate the association between risk of exposure to SARS-CoV-2 and mental health in ethnocultural groups	3273 Quebec residents June 2020	Quantitative Funding: McGill University	Exposure to the virus, COVID-19-related discrimination and stigma were associated with poorer mental health. Black participants who were exposed and discriminated against reported greater mental distress.	Interventions that take into account race and culture and that consider factors such as discrimination and historical and racial trauma are needed.
Miconi et al. (2021)^52^	Examine the extent of experiences of discrimination related to COVID-19	3273 Quebec residents June 2020	Quantitative Funding: McGill University	COVID-19-related discrimination was reported by 16.6% of participants. Non‑White participants, younger participants and healthcare workers were more likely to experience discrimination. Participants of East Asian descent and essential workers were more likely to report discrimination because of their ethnicity and occupation.	Health communication actions informed by a social pedagogy approach should target public beliefs related to the association of COVID-19 with ethnicity, age and occupation to minimize pandemic-related discrimination.
Noubicier et al. (2013)^33^	Study the meaning of aging among older Black women living in Montral	7 women from 3 sub-Saharan African countries Period: NR	Qualitative Funding: Centre for Research and Expertise in Social Gerontology	For these women, aging is inevitable. They see this period of their lives as a privilege, placing great importance on intergenerational relationships, faith and social involvement.	Social policies should take gender and ethnicity into account.
Nweze et al. (2023)^34^	Analyze the impact of racial and linguistic discrimination on mental health	531 Black Quebeckers April 2021	Quantitative Funding: Health Canada and SSHRC	English-speaking participants experienced more discrimination and reported more barriers to accessing mental health care. They also had poorer mental health than their French-speaking counterparts.	The study justifies a mechanism by which language affects mental health by exposing Black Quebeckers to more discrimination and thus higher barriers to care.
Paquette et al. (2019)^35^	Study the impact of ASD on the quality of life of mothers of Haitian origin living in Montral	12 mothers of Haitian origin of boys aged 5 to 18 with ASD Period: NR	Mixed, prospective Funding: NR	Mothers anchored their strategies for controlling ASD in their religious faith. Their quality of life was considered to be average and their coping strategies were primarily focussed on problem-solving or seeking social support. They did not confide in strangers or professionals.	Family members are the first people consulted when a physical or mental illness appears. This is why it is so important that interventions consider the subjects’ culture and choices. It is vital to develop expertise in intervening with families who have a child with ASD and on using an ethnopsychiatric approach.
Passos-Castilho et al. (2022)^67^	Identify factors associated with ICU admission and hospital mortality among COVID-19 patients	1104 patients hospitalized in Montral who tested positive for SARS-CoV-2 March to August 2020	Quantitative, retrospective Funding: Gilead, the Jewish General Hospital Foundation	Immigrants are more likely to be admitted to intensive care. This risk is higher among Caribbean Black individuals than White individuals. The risk of hospital mortality is higher among Canadian‑born individuals than among immigrants, and higher among Caribbean Black individuals (but not Africans) than White individuals.	The data highlight the disproportionate impact of COVID-19 on foreign-born communities, the significant heterogeneity of COVID-19-related outcomes within ethnic groups and between countries, and the significant gaps in understanding the individual impacts of the social determinants of health and their interactions.
Salamanca (2018)^44^	Examine the role of temporary employment agencies in Montral in exploiting immigrant labour	42 immigrant workers and members of immigrant workers’ rights groups Period: NR	Qualitative, longitudinal, participatory research Funding: NR	Nurses and attendants with valid immigration status who work through an agency are underpaid compared with workers with similar degrees and levels of education. Agency workers in the health sector, the majority of whom are Black, believe they are underpaid.	Workers with precarious immigration status experience long-term hardship, making them a vulnerable labour pool when they work for poorly regulated employment agencies. As a result, the likelihood of experiencing the effects of systemic racism is greater.
Sherry et al. (2013)^36^	Understand the attitudes and beliefs of the Haitian adult population regarding organ donation	24 Haitians 2008–2009	Qualitative Funding: Newton Foundation, MUHC Foundation	Participants recommended that Haitian health professionals and leaders use community media to spread messages about organ donation and to spark debate within families. People over 45 were more reluctant. Some of them had never heard about this issue and showed an interest in it.	The use of bilingual French/Creole Haitian moderators facilitated discussions during the study. Some of the reluctance to participate was overcome by addressing participants’ questions and concerns. The authors plan to share the study’s results with Haitian community groups, ethnocultural consultants and the Haitian media.
Spence et al. (2014)^55^	Study the effect of age at time of circumcision and race on prostate cancer risk	1590 cases 1618 matched controls Montral 2005–2009	Quantitative Funding: Canadian Cancer Society, Cancer Research Society, FRQ—Sant	Circumcision proved protective among men who were circumcised at age 36 and over (OR = 0.55). The strongest protective effect of circumcision was recorded in Black men (OR = 0.40; *p* = 0.02).	There is an inverse relationship between prostate cancer risk and circumcision performed at age 36 y and over. The protective effect appears only among Black men, the group most at risk of prostate cancer.
St-Louis et al. (2014)^37^	Study antigens in red blood cells from Black donors living in Quebec	1476 Black donors Quebec 2009–2012	Quantitative Funding: Hma‑Qubec	Genotyping results predicted the presence of rare phenotypes.	Black donors, with or without a rare phenotype, are precious to the patient cohort depending on blood transfusions and to Hma-Qubec.
Tran et al. (2013)^38^	Examine the factors leading to increased blood donation in Montral’s Black communities	33 participants, including 27 Black participants 2009–2010	Qualitative Funding: Hma‑Qubec	The participation of groups interested in bettering the lives of those affected by sickle cell anemia combined with the fact that leaders felt more comfortable donating to someone close to the community suggests that promoting blood donation from this angle would be well received.	Pointing out that phenotyped blood can be set aside for patients with sickle cell anemia is an argument for encouraging members of the Black community to donate blood. Since 2010, donors have been able to specify their ethnoracial background.
van der Ven et al. (2012)^53^	Study first-episode psychotic symptoms by ethnic background	301 patients with psychosis 2003–2010	Quantitative, prospective Funding: CIHR	Compared with the reference group (Euro‑Canadians), the African/Afro‑Caribbean group had a higher level of negative symptoms and general psychopathology scores. Ethnic groups did not differ on positive symptom scores.	Particular attention should be paid to targeting negative symptoms and improving cooperation and engagement among certain patients from racialized groups during the initial phase of psychosis.
Weiler et al. (2022)^47^	Assess vitamin D levels and modifiable factors	1035 mother-child dyads Montral 2016–2019	Quantitative, cross‑sectional Funding: CIHR	Thirty-five percent of mothers gave their consent. Most newborns had adequate vitamin D levels. However, non-White groups, particularly Black newborns, had a higher risk of deficiency (5.5 times higher).	Most newborns had adequate vitamin D levels, but 1/5 were vitamin D deficient, with differences between population groups.

***Data analysis***

We analyzed the data using a thematic approach and following the main steps involved in carrying out a research project: (1) devising participant recruitment strategies; (2) determining variables of interest to the researchers; (3) deciding on data collection and analysis methods; and (4) determining the limitations and biases inherent in studies dealing with ethnoracial background (Table3).

**Table 3:** Main characteristics of studies selected for the scoping review

Characteristic	Number of studies n (%)
Sector	
Health	23 (53.5)
Education	14 (32.5)
Social services	3 (7.0)
Employment	3 (7.0)
Year of publication	
2010–2019	22 (51.0)
2020–2024	21 (49.0)
Methods	
Quantitative	26 (60.5)
Qualitative	14 (32.5)
Mixed	3 (7.0)
Study focussed exclusively on Black populations	
Health	9 (21)
Education	3 (7)
Social services	0 (0)
Employment	0 (0)

## Results

***Selection of studies***

**Figure 1:**
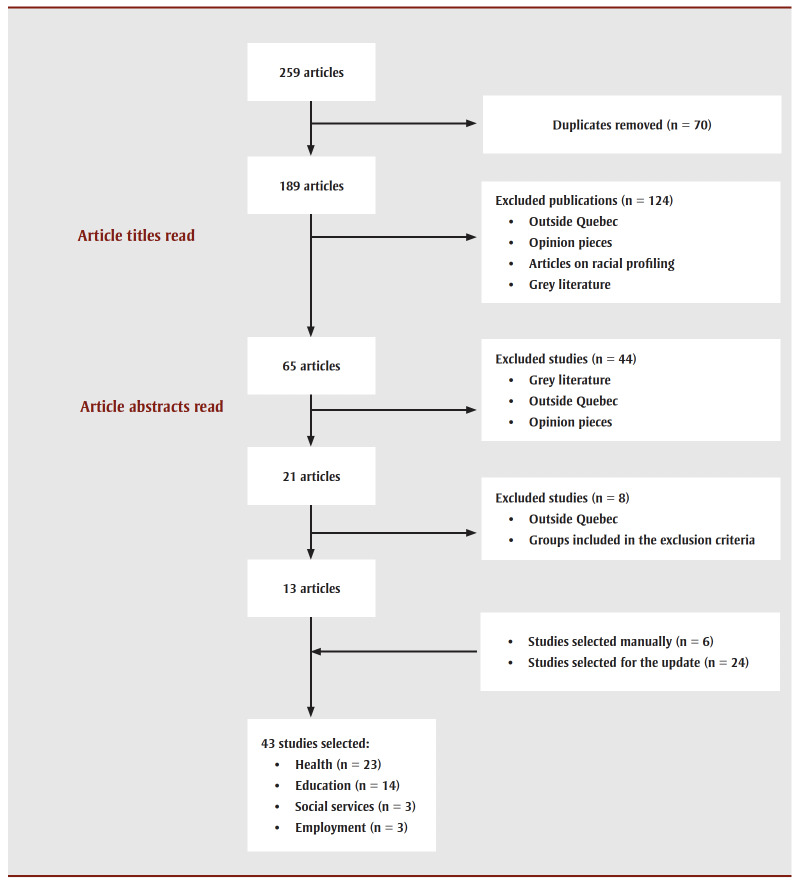
PRISMA flow diagram

The initial literature search identified 259articles from the six databases listed earlier. Once duplicates had been eliminated, 189articles remained for the two authors to review (titles, abstracts) according to the process described in the PRISMA flow diagram (Figure1). This resulted in 21articles being selected and read in full by the authors (NM and KNLN). Eight studies were excluded on the basis that they had not been carried out in Quebec, or involved groups that fell within the exclusion criteria. Following a review of the bibliographic references of the selected articles, an additional six studies were selected. An update carried out in 2024 resulted in the selection of 24additional articles, bringing the total to 43studies (Figure1).

***Study characteristics***

The studies selected relate to four sectors: health (n=23), education (n=14), social services (n=3) and employment (n=3), providing a cross-sectoral perspective on ethnoracial data collection on health and its social determinants (Table2). Descriptive, empirical or experimental studies using qualitative, quantitative or mixed methodologies were selected, with the majority being quantitative (60%). Note that 12 of the selected studies focussed exclusively on Black populations in Quebec—9 on health^30^-^38^ and 3 on education^39^-^41^ (Table3). The remaining studies relating to health, social services, education and employment were based on various ethnic groups living in Quebec, including Black communities (Table3). Only 7studies dealt with COVID-19.^50^-^52^,^56^,^66^,^67^,^69^

***Recruitment strategies***

Common recruitment methods included posters, social media, community media and participation in local events.^31^,^33^,^34^,^36^,^42^-^44^ However, recruiting individuals from specific populations with distinct characteristics (racialized populations with precarious migratory status) is more challenging owing to the difficulty in identifying them.^44^ Some authors preferred direct recruitment strategies designed to build purposeful relationships with the people contacted, such as word-of-mouth,^33^referrals from key informants within the target communities or professionals who speak the language of the people involved.^36^,^45^-^47^

Another approach involved working with community groups or students through a participatory research partnership (four studies),^31^,^41^,^44^,^48^ or leveraging the expertise of local stakeholders (representatives of community groups, religious leaders).^35^,^36^,^38^,^40^ These stakeholders’ input was especially useful in identifying Black community members as potential study participants from a database that contained no ethnoracial information.^38^

Only a few studies (n=7) provided details on the incentives used to boost participation or retention rates.^38^,^41^,^44^,^49^-^52^Incentives ranged from $0.50 to $20 for participation in a survey, and up to $50 for participation in an interview.^44^,^49^-^52^ One study reported no financial compensation for participants after taking part in a two-hour interview.^38^ In another, despite offering $50 per interview, recruiting and retaining participants proved challenging.^44^ In a participatory research study in which 20high school students were actively involved in data collection and analysis, financial compensation reached up to $500.^41^ Nevertheless, 20% of the students withdrew from this study for lack of motivation or to take on other responsibilities.^41^ The main reasons for students’ participation in the study were: wanting to make a difference in their school, and to take on the researcher’s role rather than that of the subject being observed.^41^

***Participant consent***

The process of obtaining consent was documented in 17studies, with consent typically obtained before the study began, either in writing^31^,^34^,^35^,^38^,^39^,^42^,^43^,^47^,^50^,^52^-^55^ or verbally.^37^,^40^,^49^,^54^,^56^ In one instance, consent was implied, as individuals were asked to complete a questionnaire on a voluntary basis.^57^ In another study on Haitians’ beliefs about organ donation, researchers encountered difficulties with some participants, who were reluctant to sign the consent form.^36^ Fearing that this would be used as consent to organ donation, these participants offered to sign a joint form involving all members of the focus group to avoid individual responsibility.^36^ After the researchers clarified that the consent was limited to the study and addressed participants’ concerns, the participants ultimately signed individual consent forms after the focus group.^36^ In other studies, individual consent was not required, as the research involved secondary data.^58^-^63^

In one study examining the links between exposure to SARS-CoV-2, COVID-19-related discrimination and mental health in ethnocultural groups, researchers did not disclose the true purpose of their study, given its sensitive nature. Consequently, participants’ consent was voluntary but uninformed, as the study was presented as research on COVID-19 and social distancing.^51^ In another study, researchers used a testing method that involved sending fictional resums in order to document ethnoracial discrimination in hiring.^64^

***Variables of interest to researchers***

The variables related to ethnicity, “race” and other social determinants of health collected in the selected studies are presented in Table4. Ethnoracial background and immigration data encompass a range of variables and categories (Table4). Ethnicity, whether assigned or self-reported, is defined by researchers based on biological (skin colour),^31^,^34^,^37^,^41^,^47^,^49^-^54^,^56^-^59^,^63^,^64^,^66^,^67^,^69^,^70^ geographical (country of birth or origin)^30^,^31^,^33^,^35^,^36^,^38^-^40^,^42^-^48^,^50^-^53^,^56^,^61^,^62^,^64^,^66^-^69^,^71^,^72^ or cultural (mother tongue, home language, religion) 30,31,34,39,46,51,61markers (Table4). 

**Table 4:** Variables related to ethnicity and social determinants of health identified in selected studies

Author (sample size)	Age, gender, sex	Ethnoracial background	Immigration	Socioeconomic status	Additional information
Health					
Adeponle et al. (2012)^70^ (N = 323)	Age Sex	“Race” or ethnicity (White, Black, Asian, other)	Migration status Length of stay	n/a	Mental health clinical data
Auger et al. (2012)^30^ (N = 31 868)	Age Female	Haitian community Country of birth (Haiti, other Caribbean islands, other countries, Canada)^a^ Mother tongue and language spoken at home (English, French, Creole)	n/a	n/a	Perinatal health data
Brousseau et al. (2021)^69^ (N = 2056)	Age Sex	“Race” or ethnicity (White, Black, Hispanic^b^)	n/a	n/a	COVID-19 clinical data
Carazo et al. (2022)^54^ (N = 8220)	Age Sex	“Race” or ethnicity (White, Black, other)	n/a	Type of employment	COVID-19 clinical data and psychological distress
Dagher et al. (2024)^66^ (N = 1104)	Age Sex	Country of birth Ethnicity (White, Black, Asian, Latino,^b^ Middle Eastern/North African, mixed, other)	Migration status	Material and Social Deprivation Index	Language skills (English, French) Availability of interpreters Comorbidity score
Darwish et al. (2022)^56^ (N = 150)	Age Sex	Country of origin Ethnicity (White, Black, Asian, Latino,^b^ Middle Eastern/North African, mixed, other)	Migration status	n/a	Intensive care admission, in-hospital mortality Length of hospital stay
Debrosse et al. (2024)^49^ (N = 179)	Age Sex	“Race” (Black, non-Black people of colour^b^) Ethnicity (Afro, African descent, Caribbean; Latino,^b^ Hispanic,^b^ Peruvian or Maya; Arab, Lebanese or Middle Eastern; Algerian, Kabyle, Maghrebin; Indigenous)	n/a	Median family income Unemployment rate	Neighbourhood characteristics Social identity Future aspirations Well-being (having basic needs met)
Fang et al. (2023)^31^ (N = 531)	Age Gender	Black communities in Quebec Language spoken Country of birth (participants, parents)	Generational status	Employment status Family income	Mental and physical health Access to healthcare Experiences of discrimination
Frounfelker et al. (2022)^50^ (N = 3183)	Age Gender	“Race” or ethnicity (White, East Asian, South Asian, Southeast Asian, Black, Arab, other)	n/a	Family status Income Type of employment	Mental health data Positive and negative effects of social distancing
Gomez Cardona (2012)^32^ (N = 5)	Age Female	Haitian community	Migration status Generational status Length of stay	Socioeconomic status Family status	Strategies for coping with stomach aches
Mnard et al. (2020)^63^ (N = 1387)	Age Female	“Race” (White, Black, Asian)	Migration status Country of origin Length of stay	Level of education Family status Income	Clinical data (pregnancy, nutritional intake)
Miconi et al. (2021)^51^ (N = 3273)	Age Gender	“Race” or ethnicity (White, East Asian, South Asian, Southeast Asian, Black, Arab, other) Mother tongue, religion	Generational status	Level of education Income Employment status	COVID-19 and mental health clinical data
Miconi et al. (2021)^52^ (N = 3273)	Age Gender	“Race” or ethnicity (White, East Asian, South Asian, Southeast Asian, Black, Arab, other)	Generational status	Level of education Employment status	Exposure to COVID-19 Perceived discrimination related to COVID-19
Noubicier et al. (2013)^33^ (N = 7)	Age Female	Ethnicity (sub-Saharan African)	Migration path	Socioeconomic status	Perceptions of successful aging
Nweze et al. (2023)^34^ (N = 531)	Age Gender	Black Spoken language	Generational status	Employment status Family income	Mental and physical health Experiences of discrimination Access to mental health care
Paquette et al. (2019)^35^ (N = 12)	Female Age of child	Haitian community	Migration status Generational status	n/a	Quality of life and ASD
Passos-Castilho et al. (2022)^67^ (N = 1104)	Age of mother Sex of child	“Race” or ethnicity (White, Black [Caribbean, sub-Saharan African], Asian, Latino^b^, North African/Middle Eastern, mixed, other) Country of birth	n/a	Material and Social Deprivation Index	COVID-19 clinical data Perceived discrimination
Sherry et al. (2013)^36^ (N = 24)	Age	Haitian community	n/a	Level of education	Social role or position Past experiences Beliefs about organ donation
Spence et al. (2014)^55^ (N = 3208)	Age Male	“Race” (White, Black, Asian, other)	n/a	Level of education	Clinical data (circumcision, prostate cancer screening)
St-Louis et al. (2014)^37^ (N = 1476)	n/a	Black	n/a	n/a	Genotypes
Tran et al. (2013)^38^ (N = 33)	Age Sex	Ethnicity (African, Haitian, other Caribbean)	n/a	n/a	Beliefs and attitudes about blood donation
van der Ven et al. (2012)^53^ (N = 301)	Age of mother	Statistics Canada categories (2006)^a^ Country of birth	Migration status Generational status Migration path (previous countries of residence)	Level of education	Mental health clinical data
Weiler et al. (2022)^47^ (N = 1035 pairs)	Age Female	Ethnicity according to CIHI (White, Black, East Asian, South Asian, Southeast Asian, Latino/a,^b^ Middle Eastern, other/mixed) Country of birth	n/a	Level of education Income	Lifestyle habits Vitamin D levels
Social services					
Boatswain-Kyte et al. (2020)^58^ (N = 15 875)	Age (child, mother)	White, Black, other visible minorities,^b^ unidentified	Migration status	Level of education Income Employment status Family status	Reporting, placement, legal decision Rate of inequality
Boatswain-Kyte et al. (2022)^59^ (N = 1395)	Age at placement Sex	White, Black, other visible minorities,^b^ unidentified	n/a	Socioeconomic Disadvantage Index	Type of abuse Type of placement Number of breakdowns resulting from out-of-home placement
Dufour et al. (2015)^60^ (N = 8263)	Age	“Race” or ethnicity (Black, other visible minorities,^b^ visible nonminorities^b^)	n/a	Level of education Income Family status Family size	Relocation Population density
Education					
Arcand et al. (2016)^68^ (N = 426)	Age	Country of origin	Migration status Length of stay	Employment status	Network and involvement with associations Perceived degree of loneliness and exclusion
Collins et al. (2018)^39^ (N = 11)	Age Sex	Haitian (parents) Born in Quebec (students) Mother tongue	n/a	n/a	Family experiences Elementary and high school experiences CEGEP experiences Linguistic, cultural and geographic identity Postsecondary endeavours Employment
Kamanzi et al. (2018)^62^ (N = 20 387)	Gender	Region of birth (parents)	Generational status	Level of education (parents) Family income	Postsecondary education pathways
Kamanzi (2021)^61^ (N = 8415)	Gender	African/Caribbean, Euro‑Canadian Mother tongue	Generational status	Income	Type of school Educational delay
Kanout et al. (2014)^45^ (N = 48)	Age Sex	Country of origin (parents)	Migration path	Economic profile (disadvantaged)	Day-to-day schooling Parental supervision Use of neighbourhood resources Social integration of families
Kanout et al. (2016)^71^ (N = 32 parent-child pairs)	Age Sex	Country of origin (parents)	Migration status Generational status	Economic profile (disadvantaged)	Ethnocultural identity Relationship with school Follow-up and school project Neighbourhood life
Kanout et al. (2020)^46^ (N = 1077)	Age Gender	Region of birth Mother tongue	Migration status Length of stay Premigration context	Family situation Employment status Financial resources	Institutional life experience Learning and training experience General living conditions in Quebec
Lafortune et al. (2020)^40^ (N = 53)	Age Sex	Haitian community	Migration status	n/a	Social and family support Academic achievement
Lafortune et al. (2024)^48^ (N = 27)	Age Sex	Country of origin	Migration status Length of stay	n/a	Sociocultural integration Progress in French Commitment and interest
Leduc et al. (2021)^57^ (N = 4283)	n/a	“Race” (White, Black)	n/a	n/a	Admission to medical school
Livingstone et al. (2014)^41^ (N = 20)	Age	“Race” (Black)	n/a	n/a	Equity/diversity School climate Curriculum Academic support Extracurricular activities
Magnan et al. (2017)^43^ (N = 60)	Age Sex	Region of origin (parents)	Generational status	Financial support from parents School capital (parents)	Education pathways (four types defined)
Magnan et al. (2017)^42^ (N = 60)	Age	Region of origin (parents)	Generational status	Financial support from parents School capital (parents)	Choice of postsecondary studies Family constraints Academic constraints
Magnan et al. (2023)^72^ (N = 12)	Age Gender	Ethnicity: country of origin (father, mother)	Generational status	Level of education (father, mother)	Education pathway
Employment					
Beauregard (2020)^64^ (N = 1569)	Sex	“Race” or ethnicity (Arab, Latin American, Black) Majority/minority status	n/a	n/a	Invitation for an interview
Boudarbat et al. (2010)^65^ (N = 1875)	Age Sex	Region of origin	Generational status Migration status	Level of education Employment status Salary	Job matching aspirations
Salamanca (2018)^44^ (N = 42)	Gender Age	Country of origin	Migration status Work permit	Employment sector	Violations of labour legislation Interethnic relations in the workplace

**Abbreviations:** ASD, autism spectrum disorder; CEGEP, Collge d’enseignement gnral et professionnel (general and professional teaching college in Quebec); CIHI, Canadian Institute for Health Information; n/a, not available. 

^a^ Categories assigned by a third party. 

^b^ Terminology used in original study. 

Various ethnoracial classification systems were used, including those from Statistics Canada^53^ and the Canadian Institute for Health Information,^47^ with some systems adapted to the specific characteristics of the studies in which they were used.^38^,^42^,^43^,^46^,^49^,^51^,^52^,^56^,^58^,^59^,^62^,^64^-^67^

Immigration data were self-reported in 60% (26/43) of the studies (Table 4),^31^-^35^,^40^,^42^-^46^,^48^,^49^,^51^-^53^,^56^,^58^,^60^-^63^,^65^,^66^,^68^,^70^ with migratory status and generation status (first, second generation, etc.) being the most commonly used variables (24studies). Socioeconomic status was generally defined in a consistent manner, with level of education, income and employment status being the most frequently used variables (Table 4),^31^-^34^,^36^,^42^,^43^,^45^-^47^,^49^-^55^,^58^,^59^,^61^-^63^,^66^-^69^,^71^,^72^ alongside the Material and Social Deprivation Index.^59^,^66^,^67^

***Quantitative data collection***

Primary data collection was carried out using questionnaires either distributed online^31^,^34^,^46^,^48^,^50^-^52^,^54^,^68^,^69^ or given in person,^35^,^37^,^47^,^70^ as well as by consulting medical records^51^-^53^,^55^,^56^,^66^,^67^ and supplementing demographic information with telephone or in-person interviews.^46^,^55^,^56^,^66^-^68^ In other studies, ethnicity was assigned by a third party based on the language spoken and the country of birth of the individual and their mother or both parents.^30^,^53^,^59^

Data of interest were also extracted from various centralized databases, including those maintained by Statistics Canada,^58^-^61^ the Quebec Register of Civil Status^30^ and other Quebec public agencies (departments, youth centres, etc.),^59^-^62^,^65^,^67^ with cross-referencing of multiple datasets allowing for the inclusion of missing information on ethnoracial background or socioeconomic status.^58^-^61^

***Qualitative data collection***

Data were primarily collected through individual interviews,^32^,^33^,^38^-^40^,^44^-^46^,^71^,^72^ with occasional use of focus groups.^36^,^41^,^46^,^48^,^68^ Some interviews were conducted in familiar environments (home, school, community centres) to ensure that participants felt comfortable sharing their experiences.^35^,^38^-^40^,^71^ In one study, culturally appropriate food was offered during focus groups as a sign of respect.^36^ In this study, the participants, all from the Haitian community, were grouped by age to facilitate discussion and avoid any shyness that younger participants might feel in relation to their elders because of cultural codes.^36^ Another researcher facilitated informative workshops during which participants could share their experiences of discrimination, creating a safe space for open dialogue and knowledge-sharing.^44^

To build trust between researchers and participants, other strategies included collecting data through professionals from the same community, with an immigrant background, who spoke the same language, or in the presence of a cultural mediator.^33^,^36^,^38^,^40^,^42^,^43^,^70^ Some authors found that cultural proximity enhanced engagement, as it sparked participants’ interest in the subject matter.^36^ However, some participants were hesitant to have their interviews recorded,^40^ and researchers in some studies faced challenges in maintaining appropriate professional distance with participants, as cultural proximity sometimes blurred the boundaries of the study’s objectives.^33^

***Quantitative analysis methods***

In some studies, researchers used descriptive statistics to document discrimination or coping strategies in response to health issues.^35^,^54^,^57^,^64^,^68^ Many other researchers carried out quantitative analyses using a contextualized, intersectional approach. Multivariate or regression analyses were used to compare results with a reference group, controlling for as many variables as possible.^30^,^47^,^50^,^53^,^55^,^56^,^58^,^60^,^61^,^63^,^67^,^69^,^70^ These analyses also measured the influence of ethnicity on different variables, calculated proportional risk over time (Cox analysis) and examined the geographic distribution of variables in relation to ethnicity.^30^,^51^,^59^,^60^ Note that the reference group varied across studies, and participants could be designated as “White Canadians” or “Euro-Canadians,”^30^,^47^,^51^,^53^,^56^,^61^,^67^,^69^ “Whites,”^50^,^55^,^58^,^63^ “Canadian-born”^56^,^63^,^67^ or “Blacks.”^60^,^70^

One study used the Latent Class Analysis statistical method to identify nonapparent homogeneous subgroups within a heterogeneous population.^50^ Longitudinal surveys were used to track changes in variables or phenomena over time,^30^,^61^,^64^ allowing for the identification of trends reflecting the impact of multiple waves of immigration from populations with varying sociodemographic proﬁles.^30^

***Qualitative analysis methods***

In three studies, an intersectional approach was used to address complex notions such as the inequalities experienced by Haitian students^40^ and Black African older adult women,^33^ or the discrimination and racism experienced by temporary migrants.^44^ These issues were analyzed from an intersectional perspective, taking into account identity markers such as skin colour, ethnic origin (minority/majority status), social origin (migratory and socioeconomic status) and gender.^33^,^40^,^44^

An anthropological approach was used in another study on the experience of Haitian mothers in dealing with their children’s illness.^32^ Life stories were also used to illustrate individual educational trajectories.^42^,^43^,^72^ Four studies used inductive thematic analysis to examine the data.^35^,^36^,^38^,^46^

## Discussion

The purpose of this scoping review was to analyze the methods used to collect, analyze and disseminate health, education, social services and employment data regarding Black populations in Quebec. This review enabled us to present a comprehensive corpus of data illustrating the fields of interests of Quebec researchers, the challenges in recruiting and retaining participants from Black communities in Quebec, and the quantitative and qualitative methods of analysis used.

Few studies have examined disaggregated health data based on ethnoracial background in Quebec. Most studies included in this scoping review are cross-sectional in nature, with the exception of a few longitudinal surveys^30^,^58^,^59^,^61^ conducted on secondary datasets. These longitudinal surveys allow for analyses on large samples covering the entire population targeted by the research, thereby reducing selection bias.

Several factors can influence the accuracy and relevance of conclusions drawn from studies on health issues affecting Black populations in Quebec. Biases may be introduced at various stages, from data collection to dissemination of results, posing significant methodological challenges.

Recruiting members of Black communities remains a significant challenge in qualitative studies, whereas quantitative studies rarely report this issue. However, this does not imply that researchers have not encountered such a challenge. Soliciting participation from members of Black communities requires a greater investment of time compared with other ethnocultural groups, particularly White people of European descent.^36^

The reluctance of Black communities to participate in research is rooted in historical events in which members were exploited under the guise of science. Notable examples include the Tuskegee Syphilis Experiment in the United States (1932–1972) and the illegal blood trade in Haiti during the 1970s. Additionally, in the 1980s, the Haitian community was improperly associated with HIV by the Canadian Red Cross.^73^,^74^

These historical precedents have fostered distrust of public and health authorities among Black populations in Quebec and Canada.^74^-^76^ To address this, it is crucial to keep participants well informed about research objectives and results. However, the true purpose of a study can sometimes be difficult to disclose due to its nature.^51^,^64^ These cases, though exceptions, are governed by the Tri-Council Policy Statement on Ethical Conduct for Research Involving Humans (articles 3.7A and 10.3).^77^ More attention must be paid to research dissemination practices in Quebec. Only a few studies have identified effective knowledge transfer strategies.^36^,^41^,^48^ Providing feedback to participants and the broader community can help mitigate feelings of exploitation and encourage greater participation in future research projects.^36^

Recruitment challenges can lead to selection bias, which is difficult to correct. For instance, an online survey with a low response rate (37%)^51^,^52^ and a sample predominantly composed of college- or university-educated participants may not be representative of the broader population. Therefore, the results of the study are not applicable to populations with a lower level of education.^51^,^52^ Similar logic can be applied to the language spoken, since the studies were conducted solely in English or French, which excludes allophones. Additionally, attrition bias, which involves certain participants dropping out selectively, is a common issue in research.^36^,^41^,^44^,^47^ This bias can be estimated by comparing the characteristics of participants who remained in the study with those who left.^47^

The attribution of ethnicity is another significant issue. Analysis of the studies in this review indicates that self-identification is the standard practice, compared with third-party identification. However, this method is not exempt from potential biases (nonresponses, willingness to self-identify with the majority group), which may be difficult to measure.^64^

Missing information on ethnoracial background or socioeconomic status can be supplemented by cross-referencing the data from the census or the Quebec Register of Civil Status, which contain details such as the mother’s country of birth, language spoken at home or mother tongue.^30^,^58^,^61^ Missing data can be inferred from individuals’ country of birth and language spoken.^56^,^67^ However, according to our analysis, this imputation method is not error-free if the data are not cross-referenced with migration status and generation status. 

The characteristics that define ethnicity are not fixed; they are often poorly defined and depend on the classification systems used.^16^,^17^ Ethnicity is a flexible concept. While it differs from “race,” nationality, religion and migratory status, it can include aspects of these concepts.^78^,^79^

Some researchers have tried to account for this fluidity by differentiating, within the same ethnoracial group, between individuals who speak French or English at home and those who speak their mother tongue.^30^ This enables a more detailed analysis, highlighting the complex realities that define a given group that is neither monolithic nor static. Moreover, at the individual level, there is sometimes a discrepancy between how a person is perceived by society and how they define themselves on an ethnocultural level, a meaning that can shift with time and life circumstances. Some researchers suggest defining “race” not as a biological phenotype, but as a complex social phenomenon, or classifying populations according to socioenvironmental variables rather than “race.”^26^

Our study shows that researchers prefer one-on-one interviews over focus groups for collecting qualitative data. In focus groups, the size, composition and internal dynamics of the group, as well as the topics addressed, can greatly influence what participants say and disclose, compared with the more confidential atmosphere of one-on-one interviews.^80^ Some studies show that people are more inclined to discuss sensitive topics in one-on-one interviews than in groups,^81^-^83^ while others find the opposite.^80^,^84^ This raises confidentiality concerns, as participants in focus groups get to know each other, increasing the risk of unintentional disclosure of sensitive information. The risk is particularly critical in small, tightly knit communities.

At the analytical level, descriptive analyses carried out using an intersectional approach enable the generation of hypotheses about the processes underlying observed phenomena. Correlation studies on relevant quantitative data can test these hypotheses and measure the interaction between several variables, requiring granular data on target populations. When such data are unavailable, researchers use the Material and Social Deprivation Index^7^,^59^,^66^,^67^ to identify trends. However, the ability to establish causal links remains limited.^26^ The common practice of documenting ethnoracial differences in health without adequately explaining their basis presents a number of risks. It limits primary prevention initiatives and reinforces the idea of biological determinism linked to “race.”^26^,^85^

Additionally, processing data on individuals of mixed ethnic origin poses challenges.^47^,^56^,^66^,^67^ How can these data provide us with more information, and what further analysis can be carried out? Additionally, creating broad ethnoracial categories (Black, White, Asian, Latin American, etc.) for comparisons introduces significant heterogeneity.^47^,^51^-^53^,^56^,^57^,^60^,^67^,^69^ Therefore, the results of these studies must be interpreted carefully. Analyses based on nonstandardized ethnoracial categorization risk leading to erroneous conclusions that overlook nuances that have gone unnoticed as a result of classification bias.^26^,^79^ This could jeopardize the ability to provide culturally appropriate healthcare and services.^4^,^85^

***Strengths and limitations***

This scoping review sheds light on cross-sectoral practices (health, social services, education, employment) in the collection, analysis and dissemination of ethnoracial data relating to health and its social determinants among Black populations in Quebec. To our knowledge, this is the first scoping review to be carried out on this topic in Quebec. It analyzes the experiences of these populations, considering the intersections of ethnoracial background, age, gender and language. It also reflects the interest of researchers, physicians and other health and community professionals in issues affecting racialized populations, including Black populations in Quebec. 

However, this scoping review has several limitations. It does not take into account the grey literature in Quebec on the topic. This was explored in another component of our research, which, in addition to the scoping review, included the analysis of case studies describing best practices in Quebec for culturally adapted approaches, as well as a qualitative study on the experiences and needs of researchers whose work focusses on Black populations in Quebec. Furthermore, since our review only includes studies conducted in Quebec, the results are not generally applicable to all Black populations in Canada, particularly due to Quebec’s migratory history. 

Additionally, our literature search strategy focussed on specific fields (health, education, employment and social services), which means other potentially relevant studies may have been omitted. While there may be other methods of collecting, analyzing and disseminating health data based on ethnoracial background, their omission from this study does not invalidate the scope of our work. This study is intended to be exploratory. It provides an overview of the diversity of theoretical and methodological approaches used in Quebec to collect, analyze and disseminate ethnoracial data on health and its social determinants, and associated issues.

***Future directions***

Systematic health data collection initiatives among Black populations are still uncommon in Quebec. However, the COVID-19 pandemic has sparked growing interest among health authorities and researchers in health data based on ethnoracial background. The number of studies recorded since the start of the pandemic (in 2020) is equivalent to the number recorded between 2010 and 2019 (Table3). A reflection on best practices governing the collection, protection and use of health data concerning Black populations is necessary in Quebec, in order to better equip researchers. Additionally, a qualitative study of these populations would provide a more in-depth understanding of their needs and the challenges they face.

The COVID-19 pandemic highlighted the glaring health inequities affecting Black populations in Quebec. The lack of data on the health of these populations hinders the development and implementation of public health policies. Initiatives to collect health data from Black populations in Ontario,^10^ Nova Scotia^11^ and, more recently, Manitoba,^86^ are a step in the right direction. These efforts are fostering a dynamic that could revive the political debate on this issue in Quebec.

Finally, note that 58% (25/43) of the studies presented in this scoping review were funded in part by the researchers’ respective institutions, or reported no funding at all (Table2). Promoting research on the health of Black populations should be a priority in order to better address the challenges of inequity faced by these communities. To achieve this, increased funding is needed for this field of research.

## Conclusion

The purpose of this scoping review was to document the methods and issues involved in collecting data on health and its social determinants for Black populations in Quebec. This review has enabled us to analyze current practices in Quebec using a cross-sectoral approach, with studies covering health, social services, education and employment. It also highlights the importance of collecting granular data on racialized groups, particularly on Black populations in Quebec, to support public policies designed for these populations and to promote health equity.

## Acknowledgements

This scoping review was made possible by a grant from the Public Health Agency of Canada for the project entitled Approches culturellement adaptes et accessibles pour dfinir, mesurer, analyser et rendre compte des rsultats de sant et de ses dterminants sociaux dans les communauts noires du Qubec [Culturally adapted and accessible approaches to define, measure, analyze and report on health outcomes and social determinants in Black communities in Quebec].

## Conflicts of interest

The authors declare that there are no conflicts of interest in relation to this work.

## Authors’ contributions and statement

NM, KNLN: conceptualization, data curation, formal analysis. 

NM: writing—original draft.

NM, KNLN: writing—review and editing.

The content and views expressed in this article are those of the authors and do not necessarily reflect those of the Government of Canada.
